# Nimble Gerontological Interprofessional Education During a Pandemic

**DOI:** 10.1093/geroni/igaa057.3486

**Published:** 2020-12-16

**Authors:** Megan Thomas Hebdon, Christina Wilson, Katherine Bernier Carney, Jacqueline Telonidis, Sue Chase-Cantarini

**Affiliations:** 1 University of Utah, Blacksburg, Virginia, United States; 2 University of Utah, Salt Lake City, Utah, United States

## Abstract

To improve communication and collaboration among health professionals, interprofessional education (IPE) experiences have been offered to students through the Utah Geriatric Education Consortium (UGEC) with the support of long-term care (LTC) partners since 2017. The COVID-19 pandemic presented a unique challenge in delivering in-person IPE training. Here we describe adaptations and student outcomes with our Spring/Summer 2020 training sessions. Students (n=46) from health profession programs were recruited and enrolled in the sessions. A LTC partner helped plan two-hour remote training sessions to introduce students to current issues and health care team member roles in LTC. Moderated small group discussions regarding the 4 Ms Framework and a patient case were completed using virtual breakout rooms. A shared virtual document was used to guide discussions and record insights. Student participants (n=46) were primarily White (85%), female (70%), and enrolled in physical therapy (28%), nutrition (33%), and medicine (15%) programs. Thirty-one students completed post-course satisfaction surveys with Likert-scale and open-ended questions. Most students who completed the survey agreed or strongly agreed that the course was effective (85%) and engaging (81%), and will improve care (88%). Positive course aspects included: comprehensive information with speaker experiences and use of 4 Ms; course structure with moderated small groups; and interprofessional collaboration with common goals and multiple perspectives. Despite the challenges of COVID-19, an IPE experience was effectively delivered using video conferencing technology, community collaboration, and moderated small group discussions. The successes of this IPE delivery model will enhance engagement and accessibility of future gerontological workforce training.

